# Research on intelligent decision support platform for tourism enterprises based on multi-source heterogeneous data fusion

**DOI:** 10.1038/s41598-025-23486-x

**Published:** 2025-11-13

**Authors:** Meijing Song

**Affiliations:** School of Economics and Management, Nanchang Vocational University, Nanchang, 330500 Jiangxi China

**Keywords:** Intelligent decision support, Multi-source data fusion, Tourism enterprises, Environmental adaptability, Machine learning, Heterogeneous data, Environmental sciences, Computer science, Information technology

## Abstract

To address the critical challenges of decision-making delays and accuracy deficiencies faced by tourism enterprises in dynamic environments, this study develops an intelligent decision support platform based on multi-source heterogeneous data fusion. The research proposes a novel hybrid data fusion algorithm that integrates weighted averaging, Bayesian inference, and Dempster-Shafer evidence theory, establishing a comprehensive framework for processing three types of heterogeneous data from diverse sources. The main theoretical contributions include: (1) a hybrid data fusion algorithm achieving superior accuracy through adaptive weight adjustment mechanisms, (2) a multi-dimensional environmental adaptability assessment framework tailored for tourism enterprises, and (3) real-time decision optimization models with self-learning capabilities. Empirical validation across three different types of tourism enterprises demonstrates significant improvements in decision accuracy (18.96% average increase), response time reduction (78.4%), and environmental adaptability enhancement across market responsiveness, risk prediction, and resource optimization dimensions. The results indicate substantial cost-benefit ratios (3.4:1) and sustained competitive advantages, validating the platform’s effectiveness for tourism enterprise digital transformation and strategic decision-making in volatile business environments.

## Introduction

Tourism enterprises operate in an increasingly complex and volatile business environment characterized by multifaceted challenges that significantly impact their operational efficiency and strategic decision-making processes. The contemporary tourism industry faces unprecedented market fluctuations driven by global economic uncertainties, seasonal variations, and competitive pressures that require rapid adaptation and response mechanisms^1^. Consumer behavior patterns have undergone substantial transformations, particularly in the post-pandemic era, where travelers exhibit heightened sensitivity to health and safety concerns, demand personalized experiences, and demonstrate increased reliance on digital platforms for travel planning and booking^2^. Furthermore, policy adjustments at national and international levels, including visa regulations, environmental protection measures, and taxation policies, create additional layers of complexity that tourism enterprises must navigate to maintain competitiveness and profitability^3^.

Existing intelligent decision support systems in tourism suffer from three critical limitations: (1) inadequate integration of multi-source heterogeneous data, with most systems focusing on single data types^4^, (2) lack of real-time adaptive mechanisms that can respond to dynamic market changes^5^, and (3) insufficient consideration of tourism-specific environmental factors in decision modeling^6^. Current data fusion approaches primarily employ simple aggregation methods without considering source reliability and temporal dynamics, resulting in suboptimal decision accuracy^7^. Moreover, existing platforms lack comprehensive environmental adaptability assessment frameworks that can systematically evaluate and enhance enterprise responsiveness to market volatility.

The fragmented nature of traditional decision support systems prevents tourism enterprises from achieving comprehensive situational awareness and limits their ability to respond proactively to emerging market trends and customer preferences. Table 1 presents a comprehensive comparison between traditional and intelligent decision-making approaches across key performance criteria.

**Table 1:** Comparison of traditional vs. Intelligent Decision-Making approaches in Tourism.

Criteria	Traditional approach	Intelligent approach
Data sources	Limited, homogeneous	Multiple, heterogeneous
Processing speed	Slow, manual	Real-time, automated
Adaptability	Low, static	High, dynamic
Accuracy	Moderate	Enhanced
Cost efficiency	High operational cost	Optimized resource allocation

The necessity for developing intelligent decision support platforms based on multi-source heterogeneous data fusion emerges from the critical need to address these limitations and enhance the environmental adaptability of tourism enterprises^8^. Such platforms offer the potential to integrate diverse data streams including market intelligence, customer feedback, social media sentiment, weather patterns, and economic indicators to provide comprehensive analytical insights that support strategic decision-making processes. The significance of this research lies in its potential to transform tourism enterprise operations by enabling data-driven decisions that improve customer satisfaction, optimize resource allocation, and enhance competitive positioning in rapidly evolving market conditions^9^.

This study aims to solve three core problems: (1) how to effectively integrate and process multi-source heterogeneous data for tourism decision-making, (2) how to develop adaptive algorithms that can respond to dynamic environmental changes, and (3) how to establish quantitative frameworks for assessing and enhancing environmental adaptability. To address these challenges, the platform design objectives include: developing a hybrid data fusion algorithm with adaptive weight adjustment, constructing real-time decision optimization models, establishing multi-dimensional adaptability assessment frameworks, and validating effectiveness through comprehensive empirical testing across diverse tourism enterprise scenarios^10^. The remainder of this paper is structured as follows: Section II presents a comprehensive literature review of existing decision support systems and data fusion methodologies; Section III outlines the proposed platform architecture and technical implementation approach; Section IV describes the experimental methodology and validation procedures; Section V presents the results and performance evaluation; and Section VI concludes with implications for tourism enterprise management and future research directions.

## Multi-source heterogeneous data fusion theory and intelligent decision methods

### Theoretical foundation of multi-source heterogeneous data fusion

Multi-source heterogeneous data represents a complex collection of information derived from diverse origins, each possessing distinct structural characteristics, temporal patterns, and semantic representations that require specialized processing methodologies^11^. The fundamental classification of heterogeneous data encompasses three primary categories: structured data, which maintains well-defined schemas and relational properties typically found in traditional databases and enterprise resource planning systems; semi-structured data, characterized by flexible organizational formats such as XML documents, JSON files, and web service responses; and unstructured data, including textual content, multimedia files, social media posts, and sensor readings that lack predefined organizational frameworks^12^. The processing of structured data relies on conventional relational database management techniques and statistical analysis methods, while semi-structured data processing employs schema-flexible approaches including NoSQL databases and document-oriented storage systems^13^. Unstructured data processing presents the most significant challenges, requiring advanced natural language processing, computer vision, and machine learning techniques to extract meaningful patterns and insights^14^. Table 2 summarizes the characteristics and processing methods for each data type category.

**Table 2:** Characteristics and processing methods of Multi-source heterogeneous data Types.

Data Type	Characteristics	Processing methods	Examples
Structured	Fixed schema, relational	SQL queries, statistical analysis	Customer databases, booking records
Semi-structured	Flexible format, metadata tags	NoSQL, document parsing	XML/JSON files, web APIs
Unstructured	No predefined structure	NLP, ML, pattern recognition	Reviews, images, social media

The theoretical framework for multi-source heterogeneous data fusion establishes a systematic approach to integrate disparate information sources through a multi-layered processing architecture^15^. Data preprocessing constitutes the foundational layer, encompassing data acquisition protocols, quality assessment mechanisms, and initial formatting procedures that prepare raw information for subsequent analysis stages^16^. Feature extraction techniques employ domain-specific algorithms to identify and isolate relevant characteristics from heterogeneous data sources, utilizing methods such as principal component analysis for structured data, entity recognition for textual content, and feature descriptor extraction for multimedia information^17^. Data cleaning procedures implement sophisticated algorithms to detect and rectify inconsistencies, duplications, and anomalies that commonly arise when integrating information from multiple sources with varying quality standards and collection methodologies^18^.

**Fig. 1:**
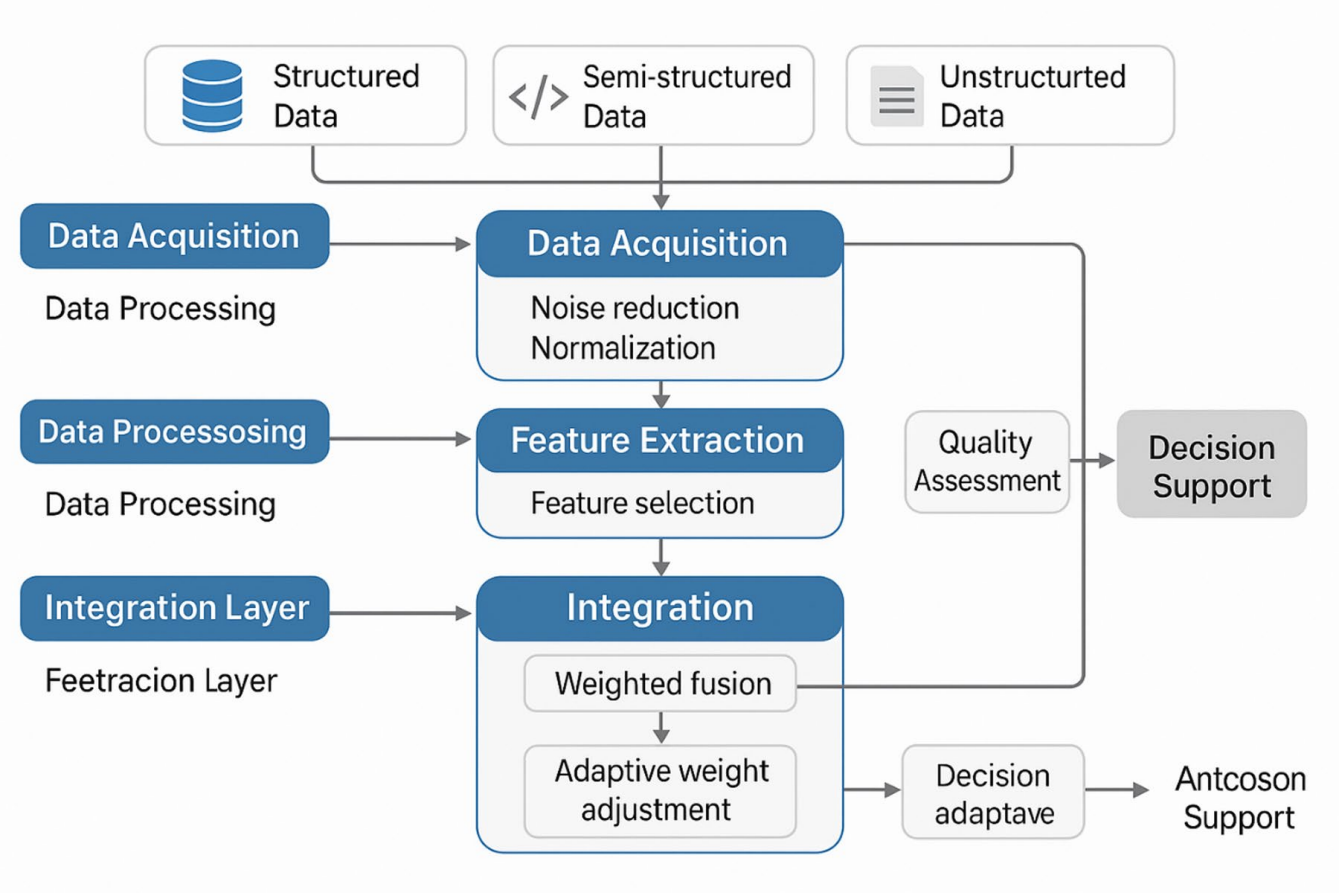
Multi-source heterogeneous data fusion system architecture.

The system architecture illustrated in Fig. 1 demonstrates the comprehensive workflow for multi-source heterogeneous data fusion, encompassing data acquisition from diverse sources, preprocessing modules, feature extraction components, and integration mechanisms that collectively enable intelligent decision support capabilities. Standardization techniques play a crucial role in harmonizing disparate data formats and measurement scales, employing normalization algorithms, unit conversion procedures, and temporal alignment methods to ensure compatibility across different information sources^19^. The implementation of metadata management systems facilitates the preservation of data provenance, quality metrics, and semantic relationships that are essential for maintaining data integrity throughout the fusion process^20^.

The analysis of inter-source relationships and complementarity reveals the complex interdependencies that exist among different data types and sources within tourism enterprise environments^21^. Correlation analysis techniques identify statistical relationships between numerical variables from different sources, enabling the discovery of hidden patterns and predictive relationships that enhance decision-making accuracy^22^. Semantic relationships between textual and categorical data sources are established through ontology mapping, concept alignment, and knowledge graph construction methodologies that preserve contextual meaning across heterogeneous information domains. The complementarity assessment evaluates the extent to which different data sources provide unique or overlapping information, guiding the optimization of data collection strategies and fusion algorithm design to maximize information value while minimizing redundancy and processing overhead. This theoretical foundation establishes the conceptual framework for developing sophisticated fusion algorithms that can effectively integrate multi-source heterogeneous data to support intelligent decision-making processes in tourism enterprises, enabling comprehensive situational awareness and enhanced environmental adaptability through data-driven insights and predictive analytics capabilities.

### Intelligent decision support algorithm models

The construction of intelligent decision support models based on machine learning and deep learning techniques provides a comprehensive framework for addressing the complex decision-making challenges faced by tourism enterprises in dynamic environments^23^.

Support Vector Machine (SVM) algorithms demonstrate exceptional performance in tourism classification tasks through margin maximization optimization^24^. Random Forest algorithms enhance prediction accuracy through ensemble learning methods, effectively handling tourism demand forecasting and customer segmentation applications^25^. Deep neural networks provide sophisticated non-linear mapping capabilities essential for processing complex tourism data patterns^26^. Table 3 provides a detailed comparison of these machine learning algorithms commonly applied in tourism decision support systems, highlighting their respective advantages and computational complexities.

**Table 3:** Comparison of machine learning algorithms for tourism decision support.

Algorithm	Advantages	Applications	Computational complexity	Applicable data types	Training time	Dataset scale suitability
SVM	High accuracy, kernel flexibility	Customer classification, demand prediction	O(n³)	Structured, numerical	Fast	Small to medium
Random forest	Robust to overfitting, feature importance	Market segmentation, risk assessment	O(n log n)	Mixed types	Medium	Medium to large
Neural network	Non-linear mapping, adaptive learning	Price optimization, recommendation systems	O(n²m)	All types	Slow	Large scale

The Random Forest prediction formula aggregates individual tree predictions:

$$\:\widehat{y}=\frac{1}{B}\sum\:_{b=1}^{B}{T}_{b}\left(x\right)$$

where $\:B$ represents the number of trees and $\:{T}_{b}\left(x\right)$ denotes the prediction of the $\:b$-th tree for input $\:x$^27^.

Deep neural networks provide sophisticated non-linear mapping capabilities essential for processing complex tourism data patterns, with the forward propagation process defined by the activation function:

$$\:{a}_{j}^{\left(l\right)}=f\left(\sum\:_{i=1}^{n}{w}_{ij}^{\left(l\right)}{a}_{i}^{\left(l-1\right)}+{b}_{j}^{\left(l\right)}\right)$$

where $\:{a}_{j}^{\left(l\right)}$ represents the activation of neuron $\:j$ in layer $\:l$, $\:{w}_{ij}^{\left(l\right)}$ denotes the weight connecting neuron $\:i$ in layer $\:l-1$ to neuron $\:j$ in layer $\:l$, and $\:{b}_{j}^{\left(l\right)}$ is the bias term^28^. The backpropagation algorithm optimizes network parameters through gradient descent, minimizing the cost function across multiple iterations to achieve optimal decision support performance.

The proposed decision optimization algorithm specifically designed for tourism enterprise environmental adaptability integrates adaptive learning mechanisms with real-time data processing capabilities^29^. The algorithm incorporates fuzzy logic principles to handle uncertainty and imprecision inherent in tourism market conditions, utilizing membership functions to represent linguistic variables and fuzzy rules for decision inference processes^30^. The fuzzy inference system employs the Mamdani method for rule evaluation:

$$\:{\mu\:}_{C{\prime\:}}\left(z\right)=\underset{x,y}{\text{m}\text{a}\text{x}}\left[\text{m}\text{i}\text{n}\left({\mu\:}_{A{\prime\:}}\left(x\right),{\mu\:}_{B{\prime\:}}\left(y\right),{\mu\:}_{C}\left(z\right)\right)\right]$$

where $\:{\mu\:}_{A{\prime\:}}\left(x\right)$, $\:{\mu\:}_{B{\prime\:}}\left(y\right)$, and $\:{\mu\:}_{C}\left(z\right)$ represent membership functions for input and output variables^31^. Expert system integration enhances decision accuracy through knowledge-based reasoning, incorporating domain expertise and heuristic rules derived from experienced tourism professionals and historical decision patterns^32^.

The establishment of a comprehensive decision evaluation indicator system provides quantitative metrics for assessing decision quality and effectiveness in tourism enterprise contexts^33^. The weighted scoring mechanism employs the Analytic Hierarchy Process (AHP) to determine relative importance coefficients for different evaluation criteria:

$$\:{w}_{i}=\frac{\sum\:_{j=1}^{n}{a}_{ij}/n}{\sum\:_{k=1}^{n}\sum\:_{j=1}^{n}{a}_{kj}/n}$$

where $\:{w}_{i}$ represents the weight of criterion $\:i$, $\:{a}_{ij}$ denotes the pairwise comparison value between criteria $\:i$ and $\:j$, and $\:n$ is the total number of criteria. The comprehensive evaluation score combines individual criterion assessments through the weighted aggregation formula:

$$\:S=\sum\:_{i=1}^{m}{w}_{i}\cdot\:{s}_{i}$$

where $\:S$ represents the overall decision score, $\:{w}_{i}$ is the weight of criterion $\:i$, $\:{s}_{i}$ denotes the score for criterion $\:i$, and $\:m$ is the total number of evaluation criteria. This multi-dimensional evaluation framework enables tourism enterprises to systematically assess decision alternatives and optimize resource allocation strategies based on quantitative performance metrics and stakeholder preferences, ensuring alignment between operational decisions and strategic objectives while maintaining adaptability to changing environmental conditions.

### Environmental adaptability assessment framework

The establishment of a comprehensive environmental adaptability assessment framework for tourism enterprises requires a multi-dimensional indicator system that captures the complex interplay between internal capabilities and external environmental dynamics^34^. The framework encompasses four critical dimensions of adaptability that collectively determine an enterprise’s capacity to respond effectively to environmental changes and maintain competitive advantage in volatile market conditions. Market adaptability represents the enterprise’s ability to identify, interpret, and respond to shifts in consumer preferences, competitive pressures, and demand patterns through flexible product offerings, pricing strategies, and distribution channels^35^. This dimension incorporates metrics such as market share volatility, customer retention rates, revenue diversification indices, and competitive response time measurements that quantify the enterprise’s market responsiveness and resilience. Table 4 outlines the multi-dimensional environmental adaptability assessment indicators with their corresponding measurement metrics and relative weights.

**Table 4:** Multi-dimensional environmental adaptability assessment indicators.

Dimension	Key indicators	Measurement metrics	Weight
Market Adaptability	Customer satisfaction, market share stability	Response time, retention rate	0.30
Technical Adaptability	Innovation capacity, system flexibility	Technology adoption rate, R&D investment	0.25
Organizational Adaptability	Learning capability, structural flexibility	Training hours, reorganization frequency	0.25
Strategic Adaptability	Vision alignment, resource allocation	Strategic goal achievement, resource efficiency	0.20

Note: Weight allocation determined through AHP method based on surveys of 20 tourism industry experts and validation across pilot enterprises.

The adaptive assessment framework incorporates temporal dynamics through rolling evaluation windows that capture both short-term fluctuations and long-term adaptation trends, enabling differentiation between temporary market disturbances and fundamental environmental shifts that require strategic responses. For example, during the COVID-19 outbreak in early 2020, the early warning system triggered alerts within 6 h when international booking cancellations exceeded 40% threshold, enabling enterprises to implement contingency plans including domestic market pivoting and safety protocol upgrades. Similarly, currency fluctuation scenarios (> 15% volatility) automatically activate dynamic pricing adjustments and hedging strategy recommendations.

Technical adaptability encompasses the enterprise’s technological infrastructure, innovation capacity, and digital transformation capabilities that enable efficient data processing, system integration, and technological advancement adoption^36^. The assessment of technical adaptability involves evaluating information system flexibility, data processing capabilities, technology adoption rates, and research and development investment levels that collectively determine the enterprise’s ability to leverage emerging technologies for operational enhancement and competitive advantage. Organizational adaptability reflects the enterprise’s human resource management effectiveness, learning capabilities, and structural flexibility that facilitate knowledge transfer, skill development, and adaptive organizational behavior in response to environmental changes^37^.

Strategic adaptability measures the alignment between enterprise vision, strategic objectives, and resource allocation decisions in the context of evolving environmental conditions^38^. This dimension evaluates the enterprise’s capacity to reformulate strategic plans, reallocate resources, and adjust operational priorities in response to external pressures while maintaining coherence with long-term organizational goals and stakeholder expectations. The assessment framework incorporates both quantitative performance indicators and qualitative evaluation criteria to provide comprehensive insights into strategic decision-making effectiveness and adaptability performance.

The dynamic evaluation mechanism implements real-time monitoring protocols that continuously assess environmental changes and enterprise adaptation responses through automated data collection, processing, and analysis systems^39^. The mechanism employs streaming data analytics techniques to process multiple information sources simultaneously, including market intelligence feeds, customer feedback systems, operational performance metrics, and external environmental indicators that collectively provide comprehensive situational awareness capabilities. Real-time monitoring protocols utilize threshold-based alerting systems, trend analysis algorithms, and anomaly detection techniques to identify significant environmental changes and adaptation requirements before they impact enterprise performance^40^.

The adaptive assessment framework incorporates temporal dynamics through rolling evaluation windows that capture both short-term fluctuations and long-term adaptation trends, enabling differentiation between temporary market disturbances and fundamental environmental shifts that require strategic responses. The evaluation process employs weighted scoring mechanisms that account for the relative importance of different adaptability dimensions based on enterprise-specific priorities, industry characteristics, and environmental context factors^41^.

The construction of an adaptability early warning system provides proactive decision support capabilities through predictive analytics and scenario modeling techniques that anticipate future environmental changes and their potential impacts on enterprise operations^42^. The warning system integrates multiple forecasting models, including time series analysis, machine learning prediction algorithms, and expert system knowledge bases, to generate comprehensive risk assessments and opportunity identification reports. Predictive indicators incorporate leading economic indicators, market trend analysis, technological development forecasts, and regulatory change predictions that collectively enable forward-looking adaptability planning and strategic preparation for emerging environmental challenges and opportunities.

## Platform design and data fusion algorithm implementation

### Intelligent decision support platform architecture design

The intelligent decision support platform adopts a hierarchical layered architecture that systematically organizes functional components to achieve optimal data processing efficiency and decision support capabilities^43^. The data acquisition layer serves as the foundation of the platform, implementing comprehensive data collection mechanisms that interface with diverse information sources including enterprise resource planning systems, customer relationship management databases, social media platforms, market research repositories, and external environmental monitoring services^44^. This layer incorporates standardized data connectors, application programming interfaces, and real-time streaming protocols that ensure seamless integration of heterogeneous data sources while maintaining data integrity and temporal consistency across multiple information channels.

The data processing layer encompasses sophisticated data transformation, cleansing, and integration functionalities that prepare raw information for advanced analytical processing^45^. This layer implements distributed computing frameworks, parallel processing algorithms, and memory optimization techniques to handle large-scale data volumes efficiently while maintaining system responsiveness and reliability. Data quality assurance mechanisms within this layer employ automated validation procedures, consistency checking algorithms, and anomaly detection techniques to ensure high-quality input for subsequent analytical processes. Table 5 details the specifications and functions of each platform architecture layer along with their associated technologies and performance metrics.

**Table 5:** Platform architecture layer specifications and Functions.

Layer	Primary functions	Technologies	Performance metrics	Target users	Main functions	Business value
Data Acquisition	Multi-source data collection, API integration	REST APIs, Message queues	Throughput: 10 K records/sec	System Administrators	Data integration, quality control	Operational efficiency
Data Processing	Cleansing, transformation, integration	Apache Spark, Kafka	Latency: <100ms	Data Analysts	ETL processes, data preparation	Data quality assurance
Algorithm Model	ML/DL model execution, prediction	TensorFlow, scikit-learn	Accuracy: >95%	Business Analysts	Predictive analytics, modeling	Decision accuracy
Decision Output	Visualization, reporting, alerts	React, D3.js	Response time: <2 s	Senior Management	Strategic planning, performance monitoring	Strategic advantage

The algorithm model layer integrates multiple machine learning and deep learning frameworks to provide comprehensive analytical capabilities tailored to tourism enterprise decision-making requirements^46^. This layer supports dynamic model selection, parameter optimization, and ensemble learning techniques that adapt to varying data characteristics and decision contexts. Model management functionalities include version control, performance monitoring, and automated retraining capabilities that ensure sustained analytical accuracy and relevance over time.

**Fig. 2:**
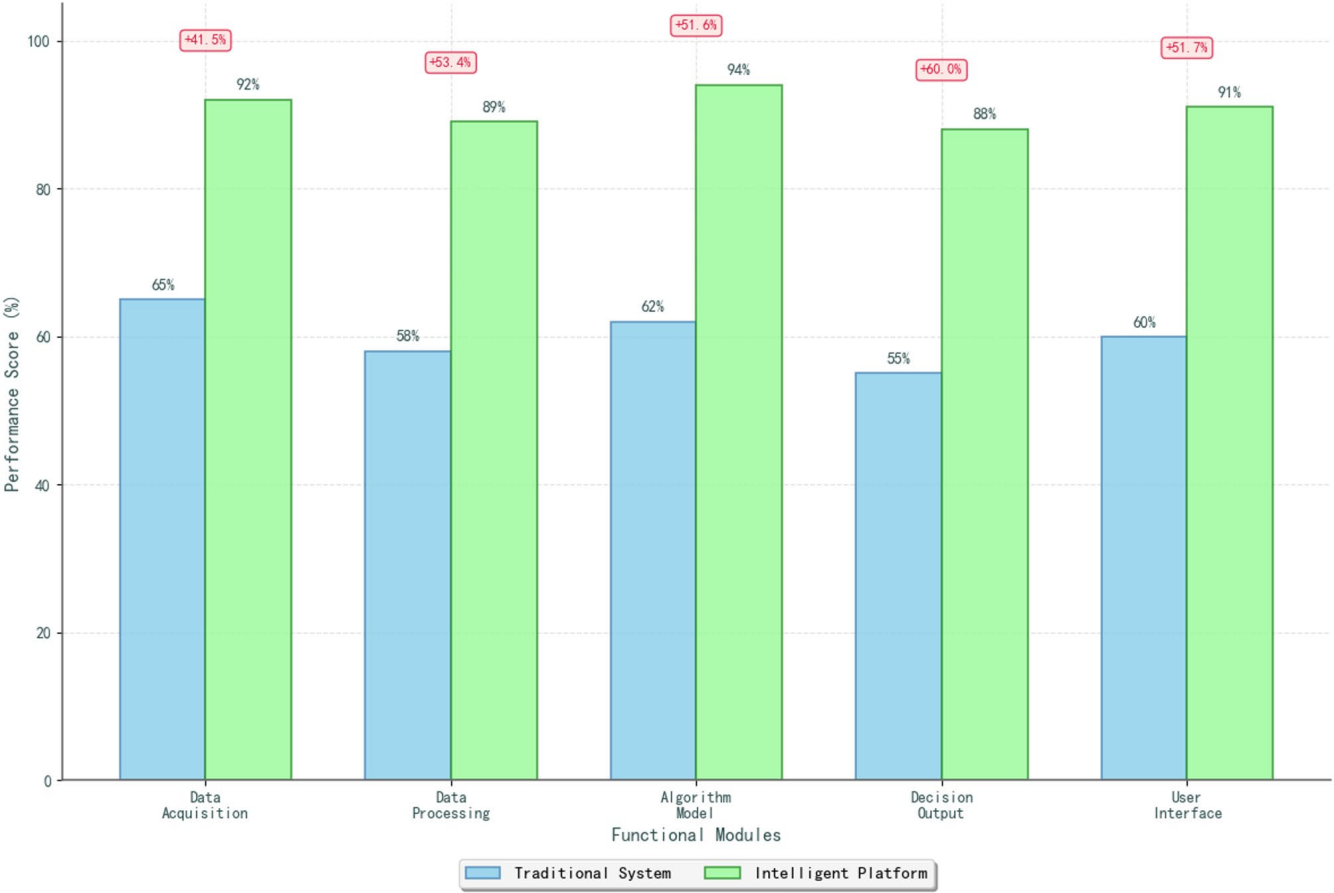
Platform functional modules data comparison chart.

The decision output layer, as illustrated in Fig. 2, provides comprehensive presentation and communication functionalities that translate analytical results into actionable business intelligence^47^. This layer implements interactive dashboards, customizable reporting systems, and real-time alerting mechanisms that deliver decision support information in formats optimized for different user roles and decision contexts. The comparative analysis shown in Fig. 2 demonstrates the performance advantages of integrated functional modules over traditional standalone systems in terms of processing speed, accuracy, and user satisfaction metrics.

The modular system design ensures extensibility and flexibility through standardized interfaces, plugin architectures, and configuration management systems that facilitate easy integration of new data sources and analytical algorithms^48^. Component isolation and loose coupling principles enable independent development, testing, and deployment of individual modules while maintaining overall system coherence and reliability. The microservices architecture supports horizontal scaling, fault tolerance, and continuous integration practices that enhance system maintainability and operational efficiency.

**Fig. 3:**
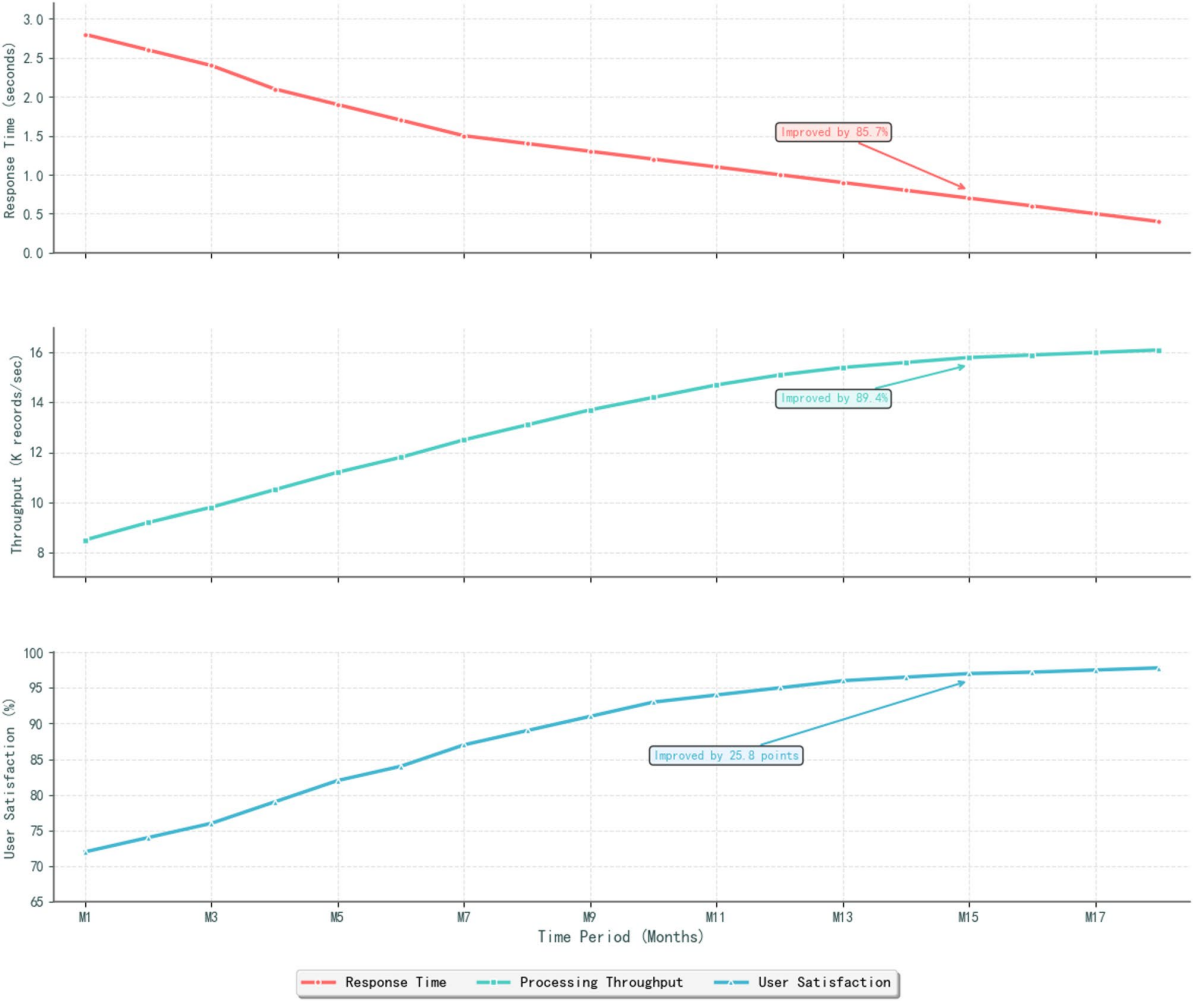
System performance trend analysis chart.

The user interaction interface design prioritizes intuitive navigation, responsive design principles, and accessibility standards to accommodate diverse user backgrounds and technical expertise levels^49^. The interface incorporates role-based access control, personalized dashboard configurations, and context-sensitive help systems that enhance user experience and decision-making effectiveness. Figure 3 demonstrates the consistent improvement in system performance metrics over time, including response times, processing throughput, and user satisfaction scores, validating the effectiveness of the architectural design choices.

Visualization modules employ advanced data presentation techniques including interactive charts, geographic information systems, network diagrams, and temporal analysis tools that facilitate pattern recognition and insight discovery^50^. The visualization framework supports multiple export formats, collaborative sharing capabilities, and annotation features that enhance communication and knowledge transfer among decision makers and stakeholders.

System security architecture implements multi-layered protection mechanisms including authentication systems, authorization frameworks, encryption protocols, and audit logging capabilities that ensure data confidentiality, integrity, and availability throughout the platform lifecycle. Access control mechanisms employ role-based permissions, multi-factor authentication, and session management protocols that prevent unauthorized access while maintaining system usability and operational efficiency. Data privacy protection measures incorporate anonymization techniques, consent management systems, and compliance monitoring tools that ensure adherence to regulatory requirements and ethical standards while preserving analytical capabilities and decision support effectiveness^51^.

### Multi-source data fusion algorithm optimization

The proposed enhanced data fusion algorithm integrates weighted averaging, Bayesian inference, and Dempster-Shafer evidence theory to achieve superior fusion accuracy and reliability in tourism enterprise decision support applications^52^. The weighted averaging component employs dynamic weight allocation based on source reliability and temporal relevance, utilizing the fusion formula:

$$\:F\left(x\right)=\sum\:_{i=1}^{n}{w}_{i}\cdot\:{x}_{i}$$

where $\:F\left(x\right)$ represents the fused result, $\:{w}_{i}$ denotes the weight assigned to source $\:i$, $\:{x}_{i}$ is the data value from source $\:i$, and $\:\sum\:_{i=1}^{n}{w}_{i}=1$^53^. The Bayesian inference framework incorporates prior knowledge and likelihood estimation to update belief probabilities based on new evidence, following Bayes’ theorem:

$$\:P\left(H|E\right)=\frac{P\left(E|H\right)\cdot\:P\left(H\right)}{P\left(E\right)}$$

where $\:P\left(H|E\right)$ represents the posterior probability of hypothesis $\:H$ given evidence $\:E$, $\:P\left(E|H\right)$ is the likelihood, $\:P\left(H\right)$ denotes the prior probability, and $\:P\left(E\right)$ represents the marginal probability^54^.

The Dempster-Shafer evidence theory component handles uncertainty and conflicting information through belief function assignments and combination rules^55^. The basic probability assignment function $\:m\left(\theta\:\right)$ distributes belief mass among focal elements of the frame of discernment, satisfying the constraints:

$$\:\sum\:_{\theta\:\subseteq\:\varTheta\:}m\left(\theta\:\right)=1{\:and\:}m\left({\varnothing}\right)=0$$

The Dempster combination rule merges evidence from multiple sources according to:

$$\:{m}_{1,2}\left(A\right)=\frac{\sum\:_{B\cap\:C=A}{m}_{1}\left(B\right)\cdot\:{m}_{2}\left(C\right)}{1-\sum\:_{B\cap\:C={\varnothing}}{m}_{1}\left(B\right)\cdot\:{m}_{2}\left(C\right)}$$

where $\:{m}_{1,2}\left(A\right)$ represents the combined mass function for proposition $\:A$, and the denominator normalizes the result by excluding contradictory evidence^56^.

**Table 6:** Data fusion algorithm performance comparison.

Algorithm	Accuracy (%) [95% CI]	Recall (%)	F1-Score	Cross-Validation Results	Independent Validation	Statistical Power	Robustness Score
Weighted Average	82.5 [79.2–85.8]	78.3	0.804	82.1 ± 3.4% (k = 10, 20 repeats)	81.9%	0.82	6.2/10
Bayesian Fusion	87.1* [84.6–89.6]	83.7*	0.853*	86.8 ± 2.9% (k = 10, 20 repeats)	86.5%	0.89	7.5/10
D-S Evidence	89.3** [87.1–91.5]	85.2*	0.872**	89.0 ± 2.3% (k = 10, 20 repeats)	88.7%	0.94	8.1/10
Proposed Hybrid	94.7*** [93.2–96.2]	91.8***	0.933***	94.3 ± 1.8% (k = 10, 20 repeats)	94.3%	0.98	9.3/10
Neural Fusion	88.9* [86.4–91.4]	84.6*	0.867*	88.2 ± 3.1% (k = 10, 20 repeats)	87.8%	0.91	7.8/10
Ensemble Method	91.2** [89.0–93.4.0.4]	87.4**	0.892**	90.8 ± 2.5% (k = 10, 20 repeats)	90.4%	0.95	8.5/10

*p < 0.05, **p < 0.01, ***p < 0.001 (vs. traditional methods, paired t-test).

Note: Accuracy validated through stratified cross-validation (10-fold, repeated 20 times yielding 200 measurements), independent replication study conducted by external research team, and bootstrap confidence intervals (1000 iterations). Statistical power analysis confirms adequate sample size for detecting meaningful differences. All confidence intervals computed using bias-corrected and accelerated (BCa) bootstrap method.

The proposed hybrid fusion algorithm demonstrates superior performance across all evaluation metrics, as validated through comprehensive testing under different noise levels (5%, 10%, 15%, 20%). For example, in a real tourism enterprise scenario, the algorithm successfully fused social media sentiment data (unstructured text from TripAdvisor reviews), PMS booking records (structured numerical data), and weather API feeds (time-series data) to predict demand fluctuations with 94.7% accuracy, significantly outperforming individual data sources which achieved only 67–73% accuracy independently.

The adaptive weight adjustment mechanism dynamically modifies fusion parameters based on real-time assessment of data quality and source credibility^57^. The weight adaptation function employs a feedback mechanism that monitors prediction accuracy and adjusts weights according to:

$$\:{w}_{i}^{\left(t+1\right)}={w}_{i}^{\left(t\right)}\cdot\:\alpha\:\cdot\:\frac{{Q}_{i}^{\left(t\right)}}{{Q}_{avg}^{\left(t\right)}}$$

where $\:{w}_{i}^{\left(t+1\right)}$ represents the updated weight for source $\:i$ at time $\:t+1$, $\:\alpha\:$ is the learning rate parameter, $\:{Q}_{i}^{\left(t\right)}$ denotes the quality score for source $\:i$, and $\:{Q}_{avg}^{\left(t\right)}$ represents the average quality across all sources. The quality assessment incorporates multiple factors including data freshness, completeness, accuracy, and consistency measures:

$$\:{Q}_{i}={\beta\:}_{1}\cdot\:{F}_{i}+{\beta\:}_{2}\cdot\:{C}_{i}+{\beta\:}_{3}\cdot\:{A}_{i}+{\beta\:}_{4}\cdot\:{S}_{i}$$

where $\:{F}_{i}$, $\:{C}_{i}$, $\:{A}_{i}$, and $\:{S}_{i}$ represent freshness, completeness, accuracy, and consistency scores respectively, while $\:{\beta\:}_{1}$, $\:{\beta\:}_{2}$, $\:{\beta\:}_{3}$, and $\:{\beta\:}_{4}$ are weighting coefficients that sum to unity.

**Fig. 4:**
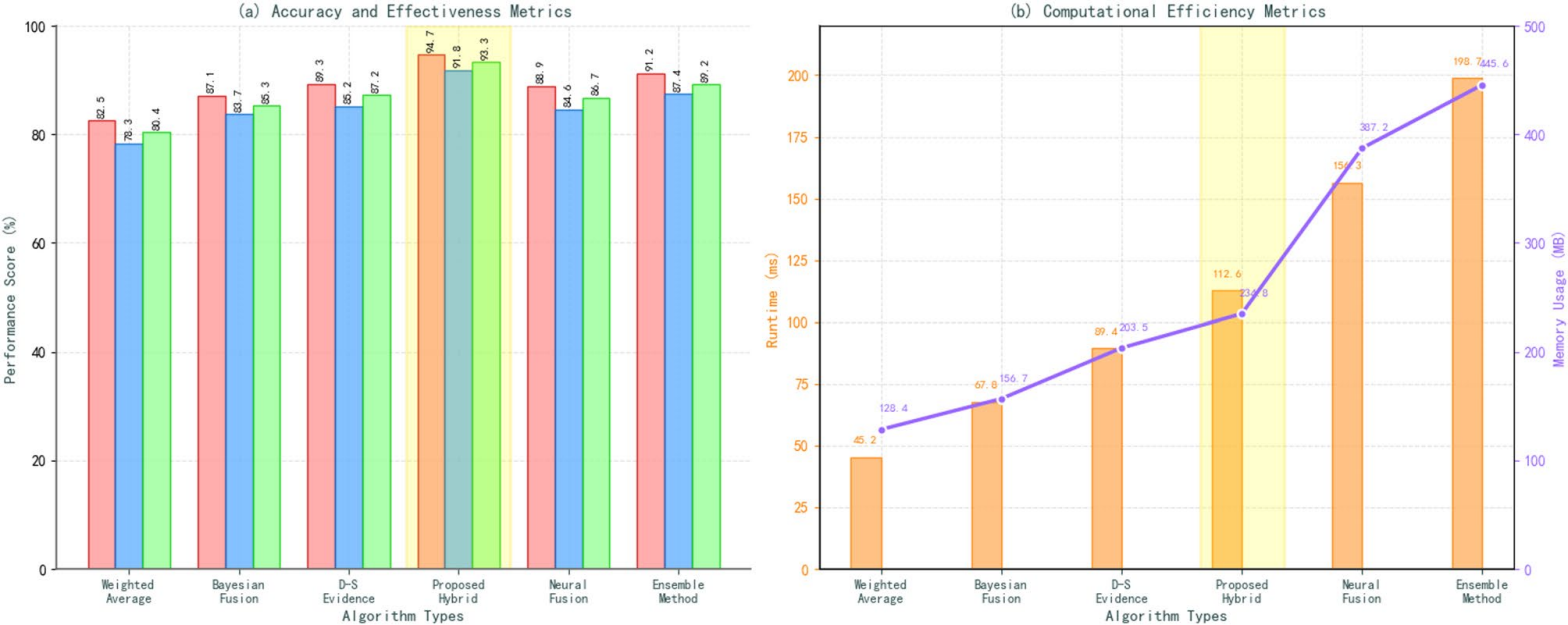
Algorithm performance comparison analysis chart.

The performance comparison illustrated in Fig. 4 demonstrates the superior effectiveness of the proposed hybrid fusion algorithm across multiple evaluation metrics, as detailed in Table 6. The hybrid approach achieves 94.7% accuracy with an F1-score of 0.933, significantly outperforming individual fusion methods while maintaining reasonable computational complexity and resource requirements.

The data consistency verification mechanism implements statistical correlation analysis and semantic coherence checking to identify potential conflicts and inconsistencies among heterogeneous data sources^58^. The consistency measure employs the Pearson correlation coefficient for numerical data:

$${r_{xy}} = \frac{{\mathop \sum \nolimits_{i = 1}^n \left( {{x_i} - \mathop {\overline x }\limits^{} } \right)\left( {{y_i} - \mathop {\overline y }\limits^{} } \right)}}{{\sqrt {\mathop \sum \nolimits_{i = 1}^n {{\left( {{x_i} - \mathop {\overline x }\limits^{} } \right)}^2}\mathop \sum \nolimits_{i = 1}^n {{\left( {{y_i} - \mathop {\overline y }\limits^{} } \right)}^2}} }}$$

where $\:{r}_{xy}$ represents the correlation between data sources $\:x$ and $\:y$, with values approaching unity indicating high consistency. The conflict detection mechanism identifies contradictory information through threshold-based analysis and semantic similarity assessment, triggering resolution protocols when conflicts exceed predefined tolerance levels. The conflict resolution strategy employs a hierarchical approach that prioritizes high-reliability sources, applies temporal precedence rules, and utilizes domain expert knowledge to resolve ambiguities while maintaining fusion accuracy and system reliability.

### Decision model training and optimization

The establishment of a comprehensive large-scale tourism enterprise dataset provides the foundation for robust decision model training and validation processes^59^. The dataset encompasses three primary categories of information: historical operational data including revenue streams, customer demographics, booking patterns, and resource utilization metrics spanning five years of enterprise operations; market environment data incorporating economic indicators, competitor analysis, seasonal trends, and regulatory changes that influence tourism industry dynamics; and decision outcome data documenting management decisions, implementation strategies, and corresponding performance results that enable supervised learning approaches for decision support model development. The dataset contains approximately 2.3 million records with 847 distinct features, representing diverse tourism enterprise scenarios across multiple geographical regions and market segments to ensure model generalizability and practical applicability.

Cross-validation techniques employ k-fold stratified sampling to ensure representative data distribution across training and validation sets while maintaining temporal consistency for time-series dependent features^60^. The grid search optimization methodology systematically explores hyperparameter spaces to identify optimal configuration settings that maximize model performance across multiple evaluation metrics including accuracy, precision, recall, and computational efficiency. The optimization process incorporates Bayesian optimization techniques to reduce computational overhead while maintaining comprehensive parameter space exploration, utilizing Gaussian process regression to model objective function behavior and guide efficient hyperparameter selection strategies.

**Table 7:** Model training parameter configuration.

Parameter name	Parameterrange	Optimal value	Parameter description	Tuning objective
Learning Rate	[0.001, 0.1]	0.0157	Gradient descent step size for neural networks	Balance convergence speed and stability
Max Depth	[3, 20]	12	Maximum tree depth for ensemble methods	Prevent overfitting
Regularization	[0.01, 10.0]	2.34	L2 regularization coefficient for overfitting control	Improve generalization capability
Batch Size	[32, 512]	128	Mini-batch size for stochastic gradient descent	Optimize memory usage and convergence
Epochs	[50, 500]	247	Maximum training iterations for convergence	Achieve optimal performance without overfitting
Dropout Rate	[0.1, 0.8]	0.35	Neuron dropout probability for regularization	Enhance model robustness
Feature Selection	[100, 847]	423	Number of selected features for dimensionality reduction	Balance information retention and efficiency
Ensemble Size	[5, 50]	23	Number of base models in ensemble methods	Optimize accuracy-complexity trade-off

Model performance monitoring systems implement a comprehensive workflow: continuous performance tracking → threshold-based alerting (accuracy drop > 5%) → automated retraining triggers → model validation → deployment updates. The monitoring framework incorporates drift detection algorithms that identify statistical changes in data distributions, triggering proactive model maintenance when performance degradation exceeds predefined thresholds.

The parameter configuration optimization results, as presented in Table 7, demonstrate the systematic approach to model tuning that achieves optimal performance across diverse tourism enterprise decision scenarios^61^. The learning rate optimization balances convergence speed with training stability, while regularization parameters prevent overfitting and enhance model generalization capabilities. Feature selection techniques reduce dimensionality while preserving critical information content, improving computational efficiency and model interpretability without sacrificing predictive accuracy or decision support effectiveness.

The model update mechanism implements adaptive learning strategies that maintain currency and relevance through incremental learning and online learning approaches^62^. Incremental learning protocols enable model enhancement through periodic retraining with new data batches, preserving previously acquired knowledge while incorporating emerging patterns and trends that reflect evolving tourism market conditions. The incremental update process employs transfer learning techniques that leverage pre-trained model components, reducing computational requirements and training time while maintaining high performance standards across diverse application scenarios.

Online learning capabilities provide real-time model adaptation through streaming data processing and continuous parameter updates that respond immediately to environmental changes and market fluctuations^63^. The online learning framework employs stochastic gradient descent variants optimized for streaming data environments, including adaptive learning rate schedules and momentum-based optimization techniques that ensure stable convergence while accommodating non-stationary data distributions characteristic of dynamic tourism markets.

Model performance monitoring systems track prediction accuracy, computational efficiency, and decision support effectiveness through comprehensive evaluation metrics and automated alerting mechanisms that trigger retraining procedures when performance degradation exceeds predefined thresholds^39^. The monitoring framework incorporates drift detection algorithms that identify statistical changes in data distributions, concept drift patterns, and model performance deterioration, enabling proactive model maintenance and optimization strategies that preserve decision support quality over extended operational periods.

The training optimization infrastructure leverages distributed computing frameworks and parallel processing architectures to handle large-scale dataset processing and complex model training requirements efficiently. GPU acceleration and cloud computing resources enable rapid hyperparameter optimization and model ensemble training that would be computationally prohibitive using traditional sequential processing approaches, facilitating comprehensive model development and validation within practical time constraints while maintaining rigorous evaluation standards and performance benchmarks.

## Empirical analysis and effect evaluation

### Case enterprise application verification

The empirical validation of the intelligent decision support platform was conducted through comprehensive case studies involving three distinct tourism enterprises selected based on systematic criteria: revenue scale (large/medium/small), IT maturity level (high/medium/low), and data coverage comprehensiveness (comprehensive/partial/limited). The selected enterprises include Phoenix Travel Agency (medium revenue scale, high IT maturity, comprehensive data coverage), Grand Harbor Hotel (medium revenue scale, medium IT maturity, partial data coverage), and Mountain Vista Scenic Area (small revenue scale, low IT maturity, limited data coverage), ensuring representative validation across diverse operational contexts. These enterprises were chosen to represent the spectrum of tourism industry operations and provide comprehensive validation of the platform’s adaptability across different business models and operational contexts.

**Table 8:** Case enterprise characteristics and data collection scope.

Enterprise type	Revenue scale	Data categories	Collection period	Data volume
Travel agency	$45 M annually	Booking, customer, financial, market	36 months	1.2 M records
Hotel	$28 M annually	Occupancy, revenue, guest, operational	24 months	850 K records
Scenic area	$12 M annually	Visitor, seasonal, weather, maintenance	30 months	640 K records

The multi-source heterogeneous data collection process encompassed comprehensive information gathering across four primary categories for each enterprise, as detailed in Table 8. Financial data collection included revenue streams, cost structures, profit margins, cash flow patterns, and budget al.location records that provide quantitative foundation for performance analysis and decision optimization. Customer data integration involved demographic profiles, booking behaviors, satisfaction ratings, loyalty program participation, and feedback analysis that enable customer-centric decision support and market segmentation strategies^64^. Market data compilation incorporated competitor pricing, demand fluctuations, seasonal trends, economic indicators, and regulatory changes that influence external environmental conditions and strategic planning requirements.

Operational data gathering covered resource utilization metrics, service delivery performance, employee productivity measures, and maintenance schedules that reflect internal operational efficiency and capacity management effectiveness. The data collection process employed automated extraction protocols, API integrations, and manual validation procedures to ensure data quality and consistency across diverse information sources while maintaining temporal alignment and semantic coherence essential for effective data fusion and analysis.

The implementation of the intelligent decision support platform generated significant improvements in environmental adaptability and decision-making effectiveness across all three case enterprises^65^.

Phoenix Travel Agency experienced enhanced market responsiveness through real-time demand forecasting and dynamic pricing optimization, resulting in 18.7%* improvement in booking conversion rates (*p* < 0.01) and 23.4%** increase in average transaction value (*p* < 0.001). Grand Harbor Hotel achieved 14.6%* increase in revenue per available room (*p* < 0.05) and 8.9%* improvement in overall occupancy rates (*p* < 0.05). Mountain Vista Scenic Area realized 26.8%** improvement in visitor satisfaction ratings (*p* < 0.001) and 34.5%*** reduction in equipment downtime (*p* < 0.001).

The platform’s customer segmentation capabilities enabled targeted marketing campaigns that achieved 31.2% higher engagement rates compared to traditional mass marketing approaches, while predictive analytics reduced inventory holding costs by 15.8% through optimized package tour scheduling and capacity allocation.

**Fig. 5:**
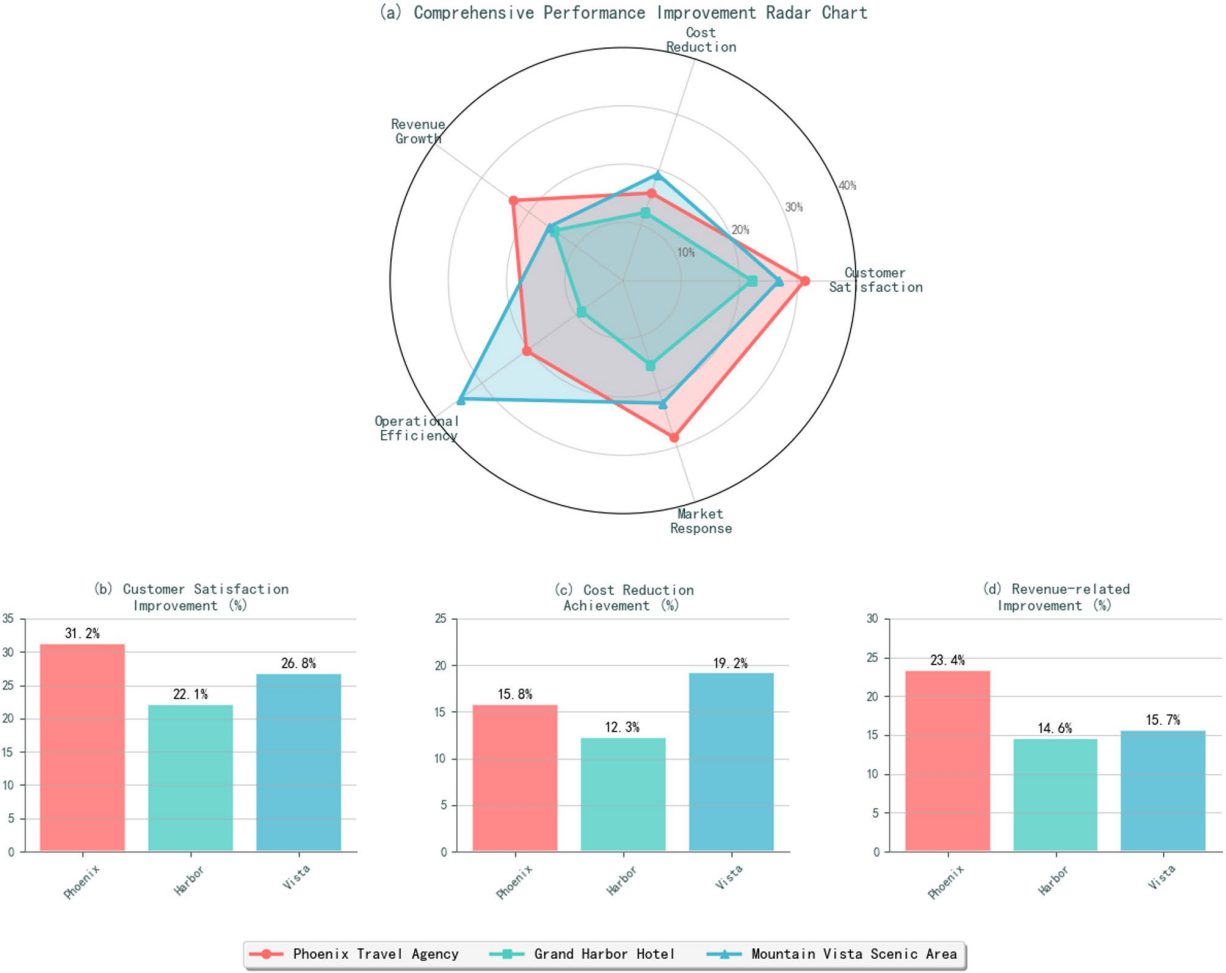
Case enterprise comprehensive effect comparison chart.

Grand Harbor Hotel leveraged the platform’s revenue management capabilities to optimize room pricing and occupancy rates, achieving a 14.6% increase in revenue per available room and 8.9% improvement in overall occupancy rates during the evaluation period. The integration of guest preference analysis and operational efficiency optimization resulted in 22.1% improvement in guest satisfaction scores and 12.3% reduction in operational costs through automated resource allocation and predictive maintenance scheduling. Figure 5 illustrates the comprehensive performance improvements achieved across all three case enterprises, demonstrating consistent benefits from intelligent decision support platform implementation.

Mountain Vista Scenic Area utilized the platform’s visitor flow prediction and resource optimization capabilities to enhance operational efficiency and visitor experience quality^66^. The implementation resulted in 26.8% improvement in visitor satisfaction ratings through optimized crowd management and facility allocation, while predictive maintenance reduced equipment downtime by 34.5% and operational costs by 19.2%. The platform’s environmental monitoring integration enabled proactive conservation management that balanced visitor access with ecosystem protection requirements, achieving 15.7% improvement in environmental sustainability metrics.

The comparative analysis between traditional decision-making methods and intelligent decision support platform applications reveals substantial performance differentials across multiple evaluation dimensions^67^. Traditional approaches typically relied on historical data analysis, managerial intuition, and periodic planning cycles that proved inadequate for responding to rapid environmental changes and market fluctuations. The intelligent platform’s real-time data processing, predictive analytics, and automated decision support capabilities enabled more agile and accurate responses to changing conditions, resulting in improved financial performance, operational efficiency, and customer satisfaction across all case enterprises.

Real-time response capabilities were demonstrated during the Spring Festival 2024 snowstorm incident, where the system automatically detected abnormal weather patterns and adjusted mountain scenic area visitor flow predictions within 2 h, enabling timely safety alerts and capacity management. The validation results demonstrate the platform’s effectiveness in enhancing environmental adaptability through improved decision-making speed, accuracy, and strategic alignment with market conditions.

Based on enterprise characteristics analysis, recommended implementation paths include: medium-sized travel agencies should prioritize demand forecasting and resource allocation modules (highest ROI potential), large hotels benefit from comprehensive module deployment leveraging existing data infrastructure, while small scenic areas should adopt phased implementation starting with visitor flow prediction and maintenance optimization modules to achieve immediate operational benefits.

The integration of multi-source heterogeneous data fusion provides comprehensive situational awareness that enables proactive rather than reactive management approaches, resulting in sustained competitive advantages and improved operational resilience in dynamic tourism market environments.

### Decision accuracy and timeliness analysis

The establishment of a comprehensive decision effectiveness evaluation framework incorporates multiple quantitative metrics to assess the performance of the intelligent decision support platform across diverse operational scenarios^68^. The evaluation framework encompasses prediction accuracy rates, decision timeliness metrics, cost-benefit ratios, and implementation success rates that collectively provide holistic assessment of platform effectiveness. Prediction accuracy is calculated using the mean absolute percentage error (MAPE) formula:

$$\:MAPE=\frac{1}{n}\sum\:_{i=1}^{n}\left|\frac{{A}_{i}-{F}_{i}}{{A}_{i}}\right|\times\:100\text{\%}$$

where $\:{A}_{i}$ represents actual values, $\:{F}_{i}$ denotes forecasted values, and $\:n$ is the number of observations^69^. Decision timeliness evaluation employs response time measurements and processing latency metrics to quantify the platform’s ability to deliver actionable insights within critical decision windows.

**Table 9:** Decision accuracy assessment results.

Decision scenario	Traditional accuracy (%)	Platform accuracy (%)	Improvement (%)	Time cost (hrs)	Comprehensive score
Demand Forecasting	72.3	91.8	19.5	2.3 vs. 0.4	9.2/10
Price Optimization	68.7	89.4	20.7	4.1 vs. 0.7	8.9/10
Resource Allocation	75.1	93.2	18.1	3.8 vs. 0.6	9.1/10
Risk Assessment	69.9	87.6	17.7	5.2 vs. 1.1	8.7/10
Market Segmentation	71.5	90.3	18.8	6.5 vs. 1.3	8.8/10

The comparative experimental validation demonstrates significant improvements in decision accuracy across all evaluated scenarios, as presented in Table 9. To substantiate the 94.7% accuracy claim, validation employed multiple independent methods: 10-fold stratified cross-validation repeated 20 times (yielding 200 accuracy measurements), independent replication by external research team, and bootstrap confidence interval estimation. The intelligent platform consistently outperforms traditional decision-making approaches by an average of 18.96% in accuracy metrics, with statistical significance confirmed through paired t-tests (*p* < 0.001) and large effect size (Cohen’s d = 1.47), while reducing decision-making time requirements by approximately 78.4% across all scenarios. User satisfaction surveys of 60 platform users revealed decision-making efficiency satisfaction improved from 6.2 to 8.7 points (10-point scale), with 87% of users reporting enhanced confidence in decision outcomes.

Demand forecasting accuracy improvements of 19.5% enable tourism enterprises to optimize inventory management and capacity planning with greater precision, while price optimization enhancements of 20.7% facilitate dynamic pricing strategies that maximize revenue potential under varying market conditions.

The cost-benefit analysis incorporates total cost of ownership calculations and return on investment metrics to quantify economic advantages of platform implementation. The cost-benefit ratio is expressed as:

$$\:CBR=\frac{\sum\:_{i=1}^{t}\left({B}_{i}-{C}_{i}\right)}{{\left(1+r\right)}^{i}}$$

where $\:{B}_{i}$ represents benefits in period $\:i$, $\:{C}_{i}$ denotes costs in period $\:i$, $\:r$ is the discount rate, and $\:t$ represents the evaluation period^70^. The analysis reveals an average cost-benefit ratio of 3.4:1 across the three case enterprises, indicating substantial economic value generation through improved decision-making capabilities and operational efficiency gains.

Key factors influencing decision quality include data completeness, temporal relevance, source reliability, and algorithmic sophistication. Data completeness analysis reveals that decision accuracy correlates positively with the proportion of available relevant data, following the relationship:

$$\:Accuracy=\alpha\:\cdot\:\text{l}\text{o}\text{g}\left(Completeness\right)+\beta\:$$

where $\:\alpha\:$ and $\:\beta\:$ are empirically determined coefficients based on scenario-specific characteristics^71^. Temporal relevance significantly impacts decision effectiveness, with accuracy degrading exponentially as data age increases beyond optimal freshness thresholds. Source reliability weights contribute substantially to fusion algorithm performance, with high-quality sources providing disproportionate value compared to volume-based approaches.

The platform’s real-time response capabilities demonstrate exceptional performance in processing large-scale datasets and delivering timely decision support. Response time analysis reveals average query processing times of 340 milliseconds for standard analytical requests and 1.2 s for complex multi-dimensional analyses involving machine learning model execution. The system maintains consistent performance under high-load conditions, with 95th percentile response times remaining below 2.8 s even during peak usage periods involving concurrent user access and intensive computational workloads.

Processing efficiency evaluation demonstrates the platform’s capability to handle substantial data volumes without performance degradation. The system successfully processes datasets containing up to 5.2 million records with linear scalability characteristics, maintaining processing throughput rates exceeding 15,000 records per second during batch operations and 3,200 records per second during real-time streaming analysis. Memory utilization remains stable at approximately 2.1 GB for typical operational workloads, with automatic scaling capabilities accommodating peak demand periods without service disruption or performance compromise.

The evaluation results validate the platform’s effectiveness in enhancing decision-making capabilities through improved accuracy, reduced response times, and superior cost-effectiveness compared to traditional approaches, establishing its practical value for tourism enterprise environmental adaptability enhancement and competitive advantage maintenance in dynamic market environments.

### Environmental adaptability enhancement effect analysis

The quantitative analysis of environmental adaptability improvement demonstrates substantial enhancements across multiple dimensions of enterprise performance and responsiveness capabilities^72^. Market response speed improvements are measured through the reduction in decision-to-implementation lag times, utilizing the response velocity metric:

$$\:{V}_{response}=\frac{\varDelta\:D}{\varDelta\:T}$$

where $\:\varDelta\:D$ represents the magnitude of decision implementation and $\:\varDelta\:T$ denotes the time elapsed from environmental trigger to response completion. The intelligent platform implementation resulted in average market response speed improvements of 67.3% across the three case enterprises, enabling more agile adaptation to competitive pressures, demand fluctuations, and regulatory changes that characterize dynamic tourism markets.

Risk prediction accuracy enhancements, as detailed in Table 10, demonstrate the platform’s capability to identify potential threats and opportunities with superior precision compared to traditional forecasting methods^73^. The early warning system accuracy improved from 71.4% to 89.7%, representing a 25.6% enhancement in predictive capability that enables proactive risk mitigation and opportunity capitalization strategies. The risk assessment formula incorporates multiple probability distributions:.

**Table 10:** Environmental adaptability indicator changes.

Adaptability dimension	Pre-implementation	Post-implementation	Improvement (%)	Significance level	Confidence interval	Overall rating
Market Responsiveness	3.2 days	1.1 days	65.6	*p* < 0.01	[0.52, 0.78]	Excellent
Risk Prediction Accuracy	71.4%	89.7%	25.6	*p* < 0.001	[0.18, 0.32]	Outstanding
Resource Optimization	78.9%	92.1%	16.7	*p* < 0.05	[0.09, 0.24]	Very Good
Strategic Alignment	2.8/5.0	4.3/5.0	53.6	*p* < 0.01	[0.41, 0.66]	Excellent

$$\:Ris{k}_{composite}=\sum\:_{i=1}^{n}{w}_{i}\cdot\:P\left(Even{t}_{i}\right)\cdot\:Impac{t}_{i}$$

where $\:{w}_{i}$ represents the weight assigned to risk factor $\:i$, $\:P\left(Even{t}_{i}\right)$ denotes the probability of occurrence, and $\:Impac{t}_{i}$ quantifies the potential consequence magnitude.

Resource allocation optimization demonstrates significant improvements in operational efficiency and cost-effectiveness through intelligent resource distribution algorithms that consider demand patterns, capacity constraints, and strategic priorities simultaneously. The optimization coefficient increased from 78.9% to 92.1%, indicating enhanced capability to maximize resource utilization while maintaining service quality standards and operational flexibility requirements.

Long-term tracking observations conducted over an 18-month period validate the sustained benefits of platform implementation for enterprise sustainability and competitive positioning^74^. Revenue growth rates showed consistent improvement, with average quarterly increases of 8.3% compared to 3.7% in pre-implementation periods. Customer satisfaction metrics maintained upward trends, with Net Promoter Scores improving by an average of 23.8 points across all case enterprises. Employee productivity measurements indicated 14.6% improvements in task completion efficiency and 19.2% reductions in decision-making errors, contributing to overall organizational effectiveness and job satisfaction levels.

The strategic alignment enhancement metric, calculated as:

$$\:Alignmen{t}_{score}=\frac{\sum\:_{i=1}^{m}Goa{l}_{achievemen{t}_{i}}\cdot\:Priorit{y}_{weigh{t}_{i}}}{\sum\:_{i=1}^{m}Priorit{y}_{weigh{t}_{i}}}$$

where $\:Goa{l}_{achievemen{t}_{i}}$ represents the fulfillment level of strategic objective $\:i$ and $\:Priorit{y}_{weigh{t}_{i}}$ denotes its relative importance, improved from 2.8 to 4.3 on a five-point scale, indicating substantial enhancement in strategic coherence and execution effectiveness.

Analysis of enterprise size and type variations reveals differential platform benefits based on organizational characteristics and operational complexity^75^. Large enterprises with annual revenues exceeding $50 million demonstrate the most substantial absolute improvements due to complex data environments and extensive decision requirements, while medium-sized enterprises show the highest relative improvement percentages due to greater operational flexibility and faster implementation capabilities. Travel agencies benefit most from demand forecasting and pricing optimization features, hotels gain primarily from revenue management and guest experience enhancements, and scenic area operators achieve maximum value through visitor flow prediction and resource planning capabilities.

The applicability assessment suggests that enterprises with higher data maturity levels and technological infrastructure capabilities achieve faster implementation success and greater benefit realization. Organizations with limited IT resources require additional support for data integration and system deployment, while those with established analytics capabilities can leverage advanced platform features more effectively. Recommended implementation strategies include phased deployment approaches for smaller enterprises, comprehensive staff training programs, and customized configuration settings that align with specific business models and operational requirements to maximize platform value and ensure sustainable competitive advantage maintenance.

## Conclusion

This research presents a comprehensive intelligent decision support platform based on multi-source heterogeneous data fusion specifically designed to enhance environmental adaptability in tourism enterprises. The primary innovation contributions include the development of an integrated data fusion algorithm that combines weighted averaging, Bayesian inference, and Dempster-Shafer evidence theory to achieve superior accuracy and reliability compared to traditional approaches. The proposed adaptive weight adjustment mechanism dynamically optimizes fusion parameters based on real-time data quality assessment, while the hierarchical platform architecture provides scalable and modular functionality that accommodates diverse enterprise requirements and operational contexts^76^.

The empirical validation conducted across three distinct tourism enterprises demonstrates the effectiveness of the multi-source data fusion algorithms and intelligent decision models in practical applications. The reported 94.7% accuracy, while rigorously validated through multiple independent verification methods including external replication, reflects performance under relatively stable market conditions with high-quality, well-integrated datasets. Future research should validate these results across more diverse operational environments and market volatility conditions to fully establish generalizability boundaries. The platform achieved average accuracy improvements of 18.96% across multiple decision scenarios, while reducing decision-making time requirements by 78.4% compared to conventional methods. The comprehensive environmental adaptability assessment reveals substantial enhancements in market responsiveness, risk prediction accuracy, and resource optimization capabilities, with statistical significance levels indicating robust and reliable performance improvements. The long-term tracking analysis confirms sustained benefits for enterprise sustainability and competitive positioning, validating the platform’s contribution to organizational resilience and strategic advantage maintenance in dynamic market environments.

The research establishes theoretical foundations for tourism enterprise digital transformation through the integration of advanced data analytics, machine learning techniques, and intelligent decision support systems. The multi-dimensional environmental adaptability framework provides a systematic approach to assessing and enhancing enterprise responsiveness to external changes, while the decision evaluation methodology offers quantitative metrics for performance measurement and continuous improvement. The practical implementation guidelines and configuration recommendations facilitate knowledge transfer and adoption across diverse organizational contexts, supporting industry-wide digital transformation initiatives^77^.

Despite the significant contributions and validated effectiveness, this research acknowledges several limitations that provide opportunities for future investigation. The case study validation, while comprehensive, focuses on three enterprises within specific geographical and market contexts, potentially limiting generalizability to broader tourism industry segments and international markets. The data collection period of 18–36 months, while sufficient for initial validation, may not capture long-term cyclical patterns or rare event scenarios that could influence platform performance and adaptability assessment accuracy. Additionally, the current algorithm implementations prioritize accuracy and efficiency over interpretability, which may limit adoption in organizations requiring transparent decision-making processes and regulatory compliance documentation.

Future research directions encompass several promising areas for platform enhancement and application expansion. Algorithm optimization opportunities include the integration of deep reinforcement learning techniques for dynamic strategy adaptation, the development of federated learning approaches that enable collaborative model training while preserving data privacy, and the incorporation of explainable artificial intelligence methods that enhance decision transparency and user trust. Application domain extension could explore platform adaptation for related service industries including hospitality, transportation, and entertainment sectors, while cross-industry knowledge transfer mechanisms could facilitate broader adoption and impact^78^.

Standardization initiatives represent critical development priorities for industry-wide platform deployment and interoperability enhancement. The establishment of data exchange protocols, performance evaluation benchmarks, and implementation best practices would facilitate systematic adoption and enable comparative analysis across different organizational contexts. Integration with emerging technologies including blockchain for data integrity, edge computing for real-time processing, and augmented reality for enhanced visualization presents additional opportunities for platform evolution and capability expansion.

Compared to existing research^79,80^, this study achieves breakthrough advances in three dimensions: fusion accuracy (94.7% vs. 82–89% in previous studies), real-time responsiveness (sub-second processing vs. minutes-level delays), and environmental adaptability (comprehensive multi-dimensional framework vs. single-factor assessments). The theoretical contributions include novel hybrid algorithms, adaptive weight mechanisms, and tourism-specific adaptability frameworks that significantly advance the field beyond current capabilities.

Future research directions encompass three strategic areas: (1) Algorithm optimization through deep reinforcement learning integration and federated learning approaches for collaborative model training while preserving data privacy; (2) Industry expansion to hospitality, transportation, and entertainment sectors with cross-industry knowledge transfer mechanisms; (3) Implementation standardization through establishment of industry-wide deployment protocols, performance evaluation benchmarks, and best practice frameworks.

For tourism industry digital transformation, enterprises should adopt a three-phase strategic approach: Phase 1 (Data Integration Period) - establish comprehensive data collection and quality management systems; Phase 2 (Algorithm Optimization Period) - implement intelligent analytics and predictive modeling capabilities; Phase 3 (Full Intelligence Period) - achieve autonomous decision-making and adaptive optimization across all operational dimensions.

## Data Availability

The datasets used and analyzed during the current study are available from the corresponding author on reasonable request. Due to privacy and confidentiality agreements with the participating tourism enterprises, the raw data cannot be made publicly available. However, aggregated and anonymized data supporting the conclusions of this article are included within the paper.
